# Imaging Plays a Key Role in the Diagnosis and Control of the Treatment of Bone Sarcoidosis

**DOI:** 10.3390/biomedicines11071866

**Published:** 2023-06-30

**Authors:** Katarzyna Błasińska, Małgorzata Ewa Jędrych, Lucyna Opoka, Witold Tomkowski, Monika Szturmowicz

**Affiliations:** 1 Department of Radiology, National Tuberculosis and Lung Diseases Research Institute, 01-138 Warsaw, Poland; lucyna.opoka@gmail.com; 2 1st Department of Lung Diseases, National Tuberculosis and Lung Diseases Research Institute, 01-138 Warsaw, Poland; e.jedrych@igichp.edu.pl (M.E.J.); w.tomkowski@igichp.edu.pl (W.T.); monika.szturmowicz@gmail.com (M.S.)

**Keywords:** bone sarcoidosis, bone marrow lesions, bone sarcoidosis treatment

## Abstract

Sarcoidosis is a multisystem granulomatous disease of unknown origin. The most frequent localizations are thoracic lymph nodes and/or parenchymal lung disease, nevertheless any other organ may be involved. Musculoskeletal sarcoidosis, previously considered a rare manifestation of the disease, is presently recognized with increasing frequency, due to the development of modern imaging modalities. The classical X-ray sign of bone sarcoidosis is the image of lace in the phalanges of the hands. Most other locations present with atypical radiological images. Therefore, they may mimic metastatic neoplastic disease, especially when they are the first sign of sarcoidosis not previously recognized. On such occasions, none of the imaging methods will give the correct diagnosis, histopathological verification, monitoring of lesions or clinical data in a patient with confirmed sarcoidosis are indicated. The article summarizes the current status of knowledge concerning the recognition and therapy of bone sarcoidosis. In addition, an illustrative case of patient with bone and bone marrow sarcoidosis is presented.

## 1. Introduction

Sarcoidosis is a rare systemic granulomatous disease of unknown etiology. It may affect multiple organs; most frequent manifestation concerns thoracic lymph nodes and/or parenchymal pulmonary disease [1]. According to The American Thoracic Society (ATS) guidelines, the key pathological features of sarcoidosis are non-necrotizing granulomas, with perilymphatic location. The differential diagnosis has to take into account all other granulomatous disorders of both infectious and non-infectious causes [1]. The most prevalent infectious causes are tuberculosis and other mycobacterial lung diseases, as well as fungal infection [1]. The non-infectious causes include sarcoid-like reaction to tumor as well as lymphoma. Many autoimmune or immune dysfunction diseases may present with granulomatous reaction, i.e., reactions to beryllium and other metals or drugs exposure [2]. Granulomas are also one of the histological features of hypersensitivity pneumonitis [3], and different forms of vasculitis (granulomatosis with polyangiitis-GPA, and eosinophilic granulomatosis with polyangiitis (EGPA)) [2]. 

Pulmonary and nodular sarcoidosis rarely causes severe symptoms, usually being a self-limited, self-curable disease in stage 1, with an increasing percentage of those who need treatment in stages 2 and 3 and causing lung fibrosis in a small proportion of patients [4].

Extra-pulmonary sarcoidosis is diagnosed in 50–74% of patients. Most life-threatening symptoms are related to cardiac sarcoidosis, and sarcoidosis-related hypercalcemia, which is usually combined with acute renal failure [1]. Another life-threatening form of sarcoidosis is neurosarcoidosis [5]. Sarcoidosis also may increase the risk of venous thromboembolic disease [6].

Musculoskeletal manifestation includes bone, muscle and joint involvement; it may present as bone lesions, arthralgia, and pathological fractures [7]. According to published data, musculoskeletal sarcoidosis is diagnosed in 25% to 33% of sarcoidosis patients [1]. Solitary bone involvement in sarcoidosis was previously considered an uncommon manifestation, affecting 3–13% of patients [8]. Nevertheless, these data may be underestimated because the osseous disease is often asymptomatic [9]. Due to the common use of nuclear magnetic resonance (MR) imaging and fluoro-deoxy-glucose positron emission tomography (FDG-PET/CT), bone and bone marrow involvement is being detected with increasing frequency. In the Netherlands study, more than one-third of FDG-PET/CT–positive sarcoidosis patients had osseous abnormalities on PET/CT, most of these lesions (94%) could not be detected on low-dose CT, and no single localization of preference was found [10].

Bone sarcoidosis may occur at any location: hands and feet, followed by the long bones of the arms and legs, pelvis, spine, and ribs. The bone involvement may be focal or diffuse and can affect one or multiple sites. Although hands and feet are considered the most common localization of bone lesions in sarcoidosis, the involvement of the axial skeleton is increasingly noted. Sparks et al. described the most frequent location of bone lesions in the spine and pelvis [11]. Zhou et al., noted in a group of 64 patients with bone sarcoidosis the localization of lesions in the spine—68.8%, in the pelvis—35.9% and in hands—15.6% [12]. Nearly half of the included patients were asymptomatic [12]. 

Imaging plays a crucial role in the diagnosis and management of bone sarcoidosis. Progress in imaging technology improved our understanding of the disease and facilitated diagnostic tissue sampling. Bone sarcoidosis usually is differentiated from bone metastatic disease, but other rare diseases, such as Blau syndrome (autoinflammatory syndrome related to the NOD2 genetic heterogeneity), should also be taken into consideration [13]. We present the comprehensive summary of imaging modalities and their performance in bone sarcoidosis. 

## 2. Characteristics of Imaging Studies Used in the Diagnostics of Bone Sarcoidosis

Diagnostic imaging modalities commonly used for musculoskeletal sarcoidosis include X-ray, ultrasonography, computed tomography, MR imaging, and FDG-PET/CT as well as other radioisotope methods. 

Imaging when musculoskeletal involvement is suspected in a patient with sarcoidosis allows the lesions to be localized and their extent and severity to be determined.

X-rays, ultrasound, computed tomography, and magnetic resonance imaging are used to assess local extension. Whole-body MR examination, PET-CT, and scintigraphy are modalities that show the extent of lesions throughout the musculoskeletal system.

Identifying bone and joint lesions in a sarcoidosis patient and implementing appropriate treatment is important because of possible complications such as pathological fractures of the bones and vertebral bodies. Imaging methods are used for follow-up during treatment.

Modalities that do not use ionizing radiation, such as magnetic resonance imaging (MRI), are particularly recommended for this purpose.

### 2.1. X-ray Imaging

X-ray remains the first-line imaging modality for the detection of bone involvement in sarcoidosis. However, it is not sensitive enough to detect early bone involvement. Therefore, the use of more sensitive imaging modalities, such as CT and MR imaging, may be necessary.

Bone sarcoidosis was first described in 1903 by Kreibich as the image of lace in the phalanges of the hands and this is considered the classic presentation of small bone involvement [8]. 

**Figure 1:**
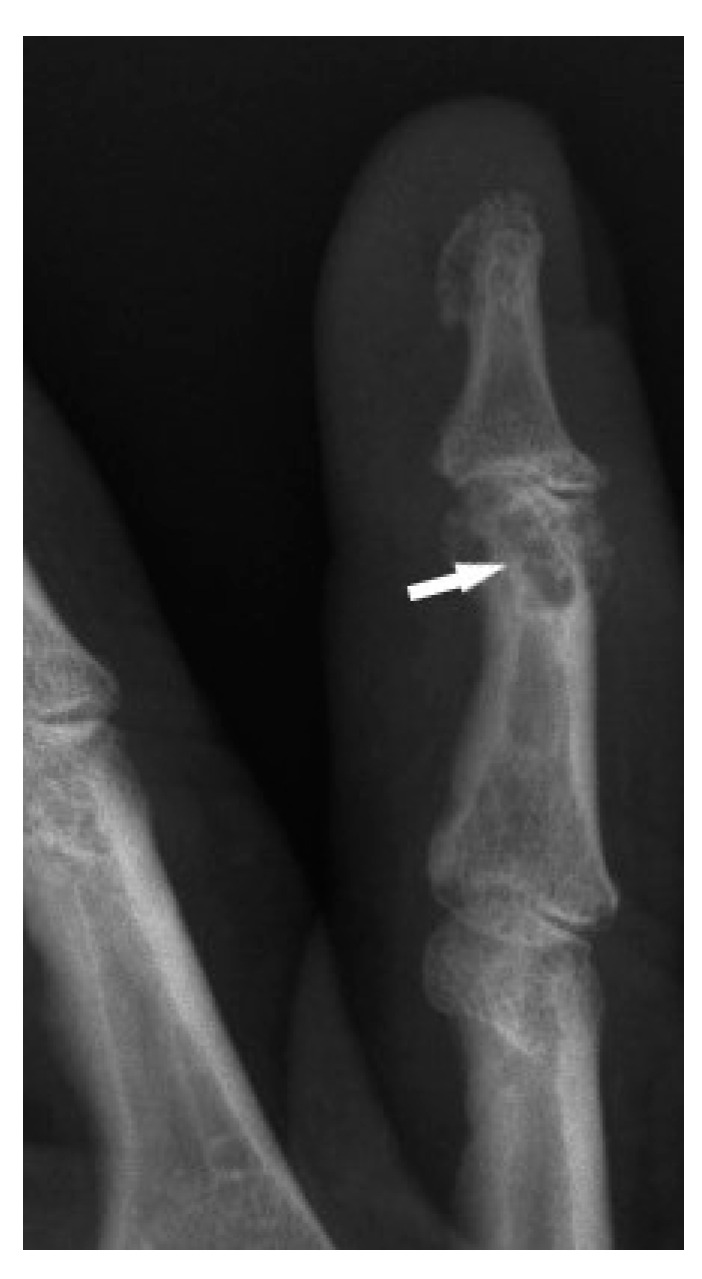
X-ray of the finger. Sarcoidosis. Cystic lesions in the middle phalanx head (white arrow).

The typical cystic X-ray pattern of bone sarcoidosis was described for small bones of the hands only (Figure 1). Other skeletal sites of sarcoidosis evaluated by this modality may be a diagnostic challenge, showing no typical radiologic features of the disease. Increasingly used modern diagnostic methods such as MRI and PET-CT indicate that multifocal bone lesions are sometimes undetectable on X-ray [10,11,12]. 

In the case of typical bony lesions of the phalanges of the hands, extending the diagnosis to MR imaging is not necessary.

### 2.2. Computed Tomography

CT is a sensitive imaging modality that can detect smaller bone lesions in sarcoidosis. It can provide detailed information on the extent and location of the bone involvement. 

**Figure 2:**
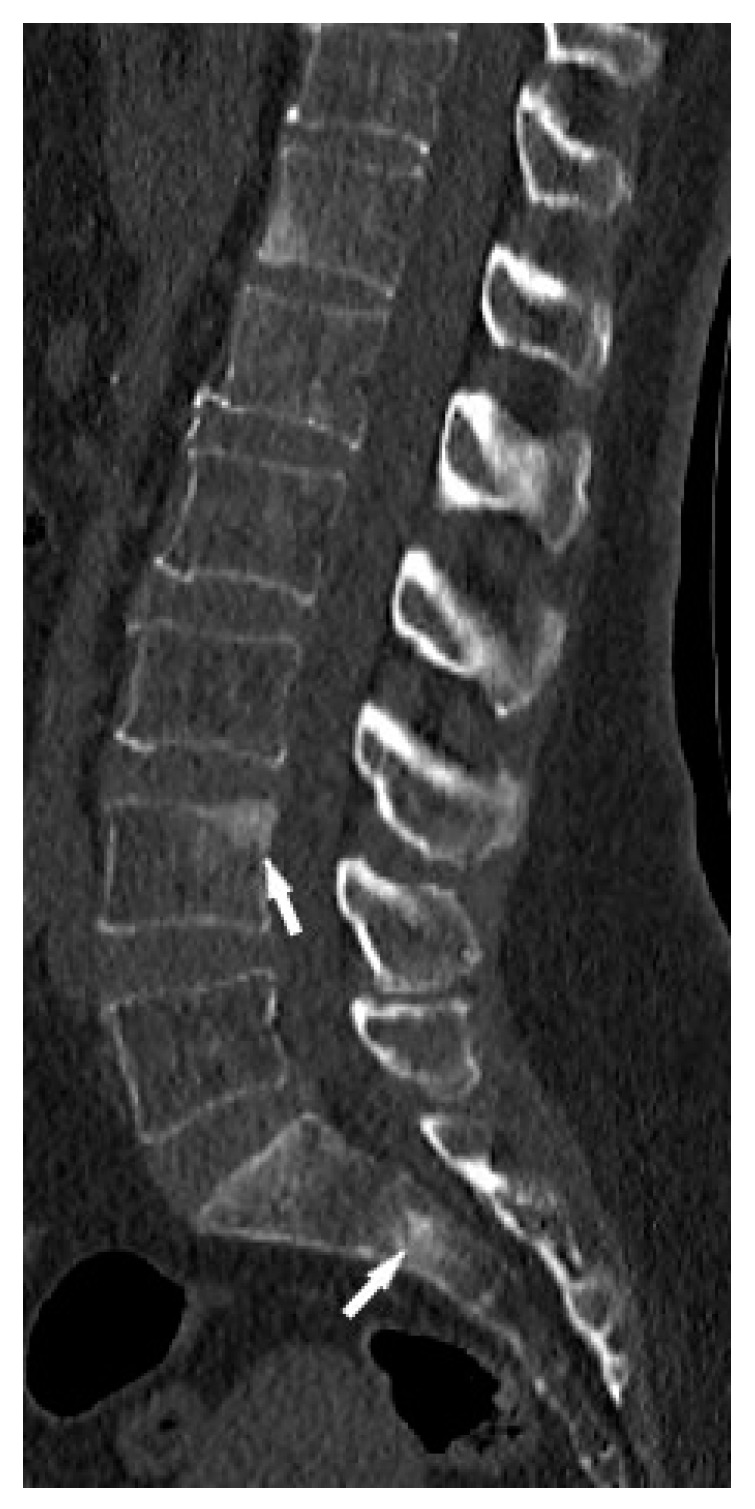
Lumbar spine CT, bone window. Osteosclerotic lesions (white arrows) in the L3 and S2 vertebral bodies.

CT is superior to X-ray in detecting bone lesions and could provide valuable information for treatment planning. The CT images categorize the bone lesions as osteolytic, osteosclerotic or mixed lytic-sclerotic (Figure 2). Evaluation of foci in the long bones and axial skeleton does not require the administration of iodine contrast agent; three-plane evaluation of bone window lesions is sufficient.

Computed tomography, although showing greater sensitivity compared to X-ray, is a less sensitive method compared to MRI, besides being associated with ionizing radiation doses.

The CT-guided biopsy of the lesions enables collection of a sample for histopathological examination. The presence of non-caseating granulomas in the collected material is often the only method of confirming the diagnosis and differentiating bone sarcoidosis from other conditions, such as metastases or multiple myeloma. 

### 2.3. Ultrasound Examination

Ultrasound examination is a useful method for evaluating joint cavity effusion in cases of acute and chronic arthritis in the course of sarcoidosis, also allowing assessment of synovium, tendons and cartilage. Under ultrasound’s guidance joint fluid can be collected for examination. This modality is also useful in visualizing sarcoid changes in soft tissues and can be used to localize and biopsy the lesions.

### 2.4. Magnetic Resonance

**Figure 3:**
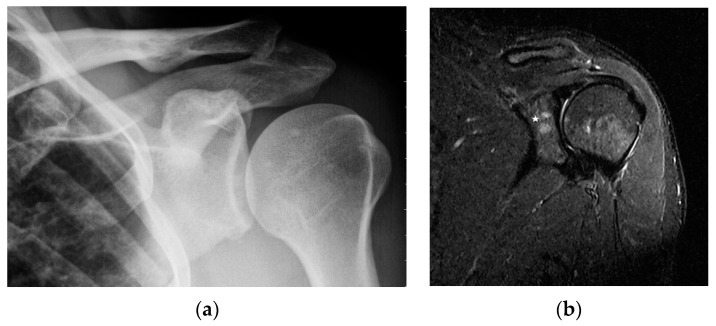
Radiograph (**a**) and magnetic resonance of the shoulder joint. T1-weighted image with fat saturation and with contrast administration (**b**). 40-year-old patient with sarcoidosis. Two contrast-enhanced lesions in the acetabulum of the joint ((**b**), asterisk) not visible on X-ray (**a**).

MR is a highly sensitive imaging modality that can detect bone marrow involvement in sarcoidosis. It also provides the detailed information on soft tissue involvement, synovitis and joint inflammation. Occasionally, MR images detect clinically and radiologically silent lesions (Figure 3a,b).

Moore et al. in a study of 42 patients with sarcoidosis showed that MR imaging detected more lesions in the long bones and axial skeleton compared to X-rays [14]. The sensitivity and specificity of routine MRI for intramedullary foci in sarcoidosis in the further Moore (2012) study were determined to be 46.3% and 94.9%, respectively, with a negative predictive value of 63.9%. The study was concerned with differentiating sarcoidosis foci from metastases on the basis of morphological features, e.g., the presence of fat in the lesion [15]. Other studies demonstrate that MRI and PET-CT have a sensitivity of 98.1% in detecting musculoskeletal lesions [12]. Despite expressing high sensitivity, MR lacks specificity. Intramedullary lesions in MR images, although clearly visible, have no characteristic patterns for sarcoidosis, and require definitive confirmation of the diagnosis by histopathological examination.

A routine MR examination protocol for the musculoskeletal system including T1-weighted, T2-weighted and STIR images should be supplemented with the administration of gadolinium contrast agent and diffusion-weighted images.

It is important to note that MR imaging does not expose the patient to ionizing radiation, so it can be used to monitor lesions under treatment.

### 2.5. Nuclear Imaging

PET/CT is a combined imaging modality that obtains the metabolic information from a PET scan and the anatomical information from a CT scan. It can be useful in detecting bone involvement in sarcoidosis, particularly in cases where other imaging modalities are inconclusive. FDG-PET/CT study allows detection of metabolically active foci in the musculoskeletal system and assessment of the extent of the disease. Although the sensitivity of PET-CT in bone sarcoidosis is sometimes reported to be 98.1% [12], specificity is limited in differentiating between sarcoidosis and metastases.

Due to its high sensitivity, the examination is useful for the evaluation of asymptomatic lesions and may be useful for sarcoidosis patients with unspecified bone complaints [12].

**Figure 4:**
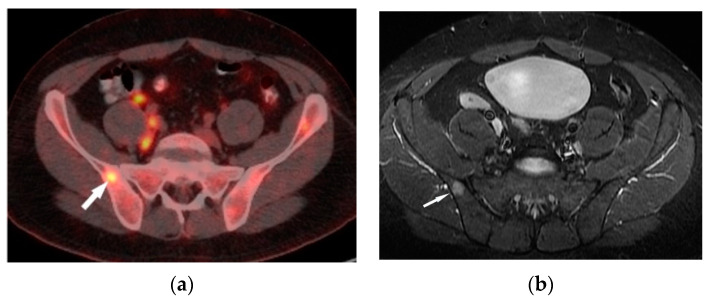
FDG-PET/CT (**a**) and T1–weighted MRI image with contrast enhancement (**b**) presents an enhanced lesion with increased FDG uptake (white arrows) in the right iliac bone.

A decrease in the FDG uptake in the follow-up PET/CT study is indicative of a metabolic response associated with regression of the sarcoid foci. A promising diagnostic tool for musculoskeletal sarcoidosis appears to be a hybrid of PET and MRI [16] (Figure 4a,b).

Gallium scintigraphy has been used for many years in the diagnosis of sarcoidosis, including those involving the musculoskeletal system. The diagnostic efficacy is enhanced by the use of single proton emission CT (SPECT) and SPECT-CT. Sarcoid lesions show increased uptake of the 67 Ga tracer. Various studies have determined the sensitivity of gallium scintigraphy in the evaluation of active lesions in sarcoidosis to be between 60 and 90% with a specificity of approximately 50% [12,17,18]. In the Zhou study, the sensitivity for detecting active sarcoidosis in histologically proven sarcoidosis was 97% for 18F-FDG-PET and 88% for 67 Ga scintigraphy [12].

When comparing FDG-PET/CT and gallium scintigraphy, it should be noted that the radiation dose in scintigraphy is three times higher and the acquisition takes place 24 h after the uptake of the radiotracer.

## 3. Imaging of Sarcoid Lesions in the Musculoskeletal System

### 3.1. Articular Involvement

Sarcoidosis-associated arthritis may be acute or chronic. Acute arthritis, diagnosed more frequently than chronic, is a self-limiting disease often diagnosed as a component of Löefgren’s syndrome [19]. In 40% of patients, it is the first manifestation of the disease, and in most of them it affects the ankle joints bilaterally. It is extremely rare for a single joint to be involved [19]. Acute arthritis is characterized by joint effusion and synovitis; tenosynovitis may also be present. In addition to pain and swelling of the joint, the characteristic symptom is erythema nodosum. As the disease resolves, no serious damage to the cartilage and articular surfaces is observed. Only swelling of the periosteal soft tissues is visible in the X-ray images. US and MRI show joint effusion and features of synovitis.

**Figure 5:**
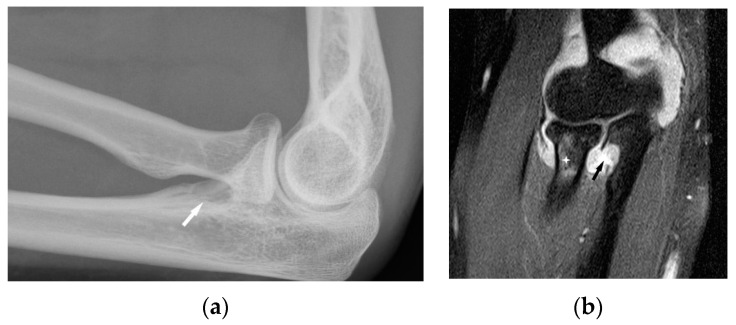
Radiograph (**a**) and MRI of the elbow (**b**) performed in a 45-year-old woman with sarcoidosis. Osteolytic lesion in the proximal part of the elbow bone ((**a**), white arrow). T2-weighted image with fat saturation revealed a synovitis, erosion in the proximal part of the elbow bone corresponding to this bone lesion (black arrow) and slight bone marrow edema in the head and neck of the radius (asterisk).

Chronic arthritis, either mono-articular or poly-articular, occurs in the later phases of the disease, usually as a part of a multisystem manifestation. In addition to synovitis (Figure 5a,b), erosions of the articular surfaces may be present, making it necessary to differentiate the lesions from reactive or rheumatoid arthritis [19]. An additional difficulty is that the rheumatoid factor may be positive in 10–47% of cases [20]. If differential diagnosis is necessary, a synovial US-guided biopsy is performed. Detection of granulomas in a synovial biopsy sample helps make the diagnosis of sarcoidosis [20]. X-ray shows soft tissue swelling and epiphyseal demineralization. 

Long-lasting joint inflammation leads to the narrowing of the joint space. In the sarcoid arthritis diagnostic imaging, we mainly use X-ray, US, and MR imaging. MRI shows with high sensitivity the joint effusion and features of synovitis, cartilage destruction, and inflammatory changes of periarticular structures such as peritendinitis, tendinitis or bursitis (Figure 5a,b) [20].

### 3.2. Bone Sarcoidosis

Only the radiological pattern of the lesions in the phalanges of the hands and feet, resembling a lace on X-ray, is described as characteristic of bone sarcoidosis.

**Figure 6:**
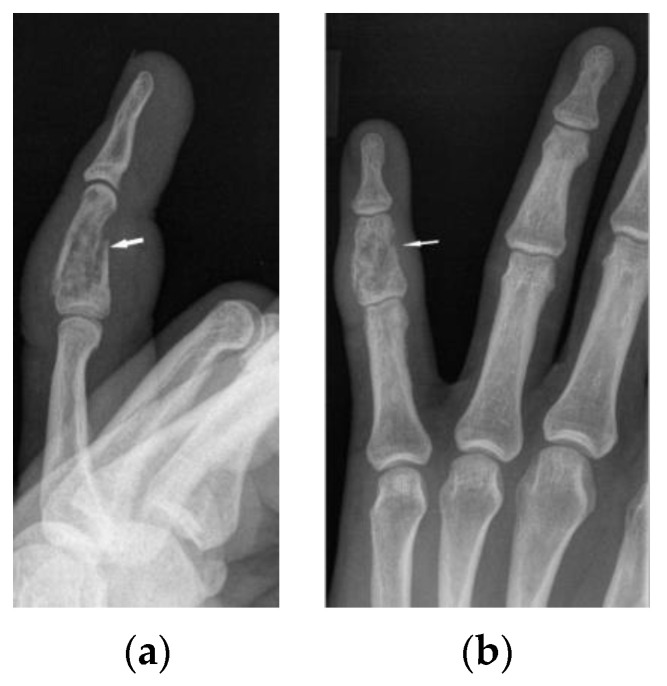
X-ray of the left hand, lateral view (**a**), PA (**b**). Osteolytic pattern in the middle phalanx with pathological fracture (white arrows). Soft tissue swelling.

**Figure 7:**
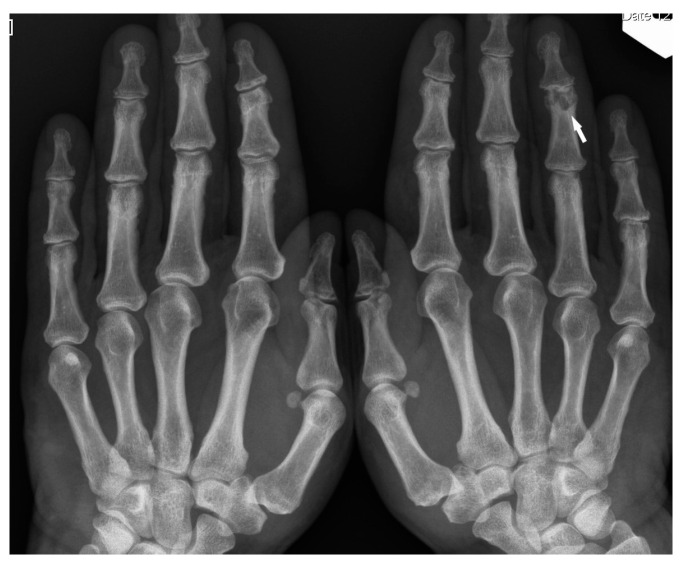
X-ray of the hands, PA. Unilateral osteolytic-cystic lesions in the subchondral layer of the middle phalanx of the 4th finger of the left hand (white arrow).

The radiographic appearance of phalanges occupied by sarcoidosis includes lytic lesions of various sizes, called cysts. Cystic lesions, mostly punched-out, may be accompanied by soft tissue nodules. The presence of large cysts increase the risk of pathological bone fracture [20] (Figure 6a,b). Numerous small cysts are more frequently observed. The articular surfaces are preserved, although cysts located in the subchondral layer might mimic erosions (Figure 7). Periosteitis is uncommon.

Features of bony destruction may be permeative and cause scalloping of the cortex, whereby cortical margins are preserved. Bone destruction with moth-eaten pattern may involve the cortex, usually with associated soft tissue swelling [19]. The cortical and trabecular architecture is usually remodeled [20]. The phalanges of the second and third fingers of the hand are most often involved, leading to the image of sausage-shaped fingers [20].

If the hand or feet bone lesions are isolated, they require differentiation from enchondroma, subchondral cysts, metastases, fibrous dysplasia or osteomyelitis.

In the long bones and axial skeleton, bone lesions can have an osteolytic, osteosclerotic or mixed appearance. Osteosclerotic lesions are more common in the spine [19]. Unlike phalanges, they do not cause disruption of the cortex or involvement of the surrounding soft tissues. 

MR imaging is a highly sensitive method for the diagnosis of bone sarcoidosis, but the lesions are not specific. Moreover, the serum hypercalcemia that may be present both in sarcoidosis and in metastatic bone disease is not helpful in differential diagnosis. 

**Figure 8:**
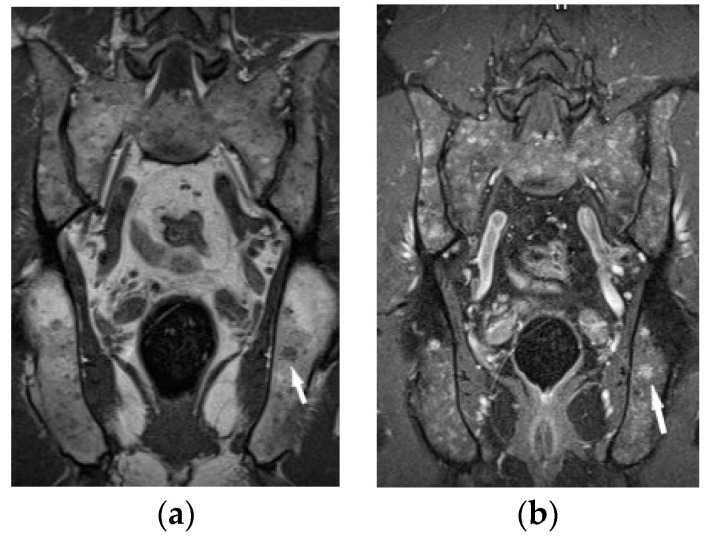
MRI of the pelvis. T1-weighted image, coronal plane (**a**), T1-weighed image with fat saturation and with contrast administration (**b**) shows multiple hypointense bone marrow lesions (**a**) with contrast enhancement ((**b**), white arrows).

Bone marrow sarcoid foci are hypointense on T1-weighted images and hyperintense on T2-weighted and STIR image, with the same appearance as metastases, multiple myeloma or lymphoma [19] (Figure 8a,b). In T2-weighted images, lesions in sarcoidosis of the bone marrow may also show low and intermediate signal intensity, with signal being higher in STIR images [15].

**Figure 9:**
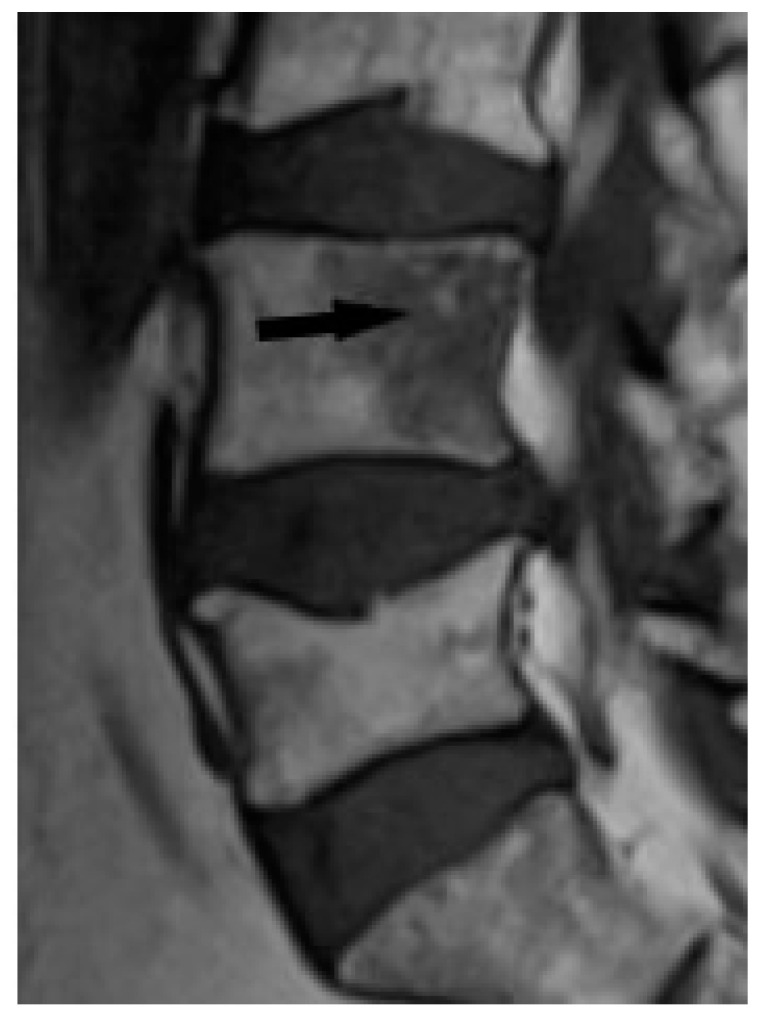
T1-weighted image. Hypointense lesion with irregular margins and with small foci of hyperintense fat (black arrow).

The outlines of the lesions can be smooth or irregular, and sometimes the presence of normal fatty bone marrow within the lesion can be seen, which was thought to be an indicator of the benign nature of the lesion [15] (Figure 9). The presence of fat in the lesion may indicate involution. An additional differentiating feature may be the presence of a mass in the soft tissues, which is extremely rare in sarcoidosis, but more common in metastases [15].

However, differentiation between sarcoidosis and metastases may be difficult on a routine MR examination protocol. Moore et al. showed in an analysis of MR images of 34 patients with metastases and spinal sarcoidosis that it is not possible to differentiate between lesions based on routine MR images alone [15].

**Figure 10:**
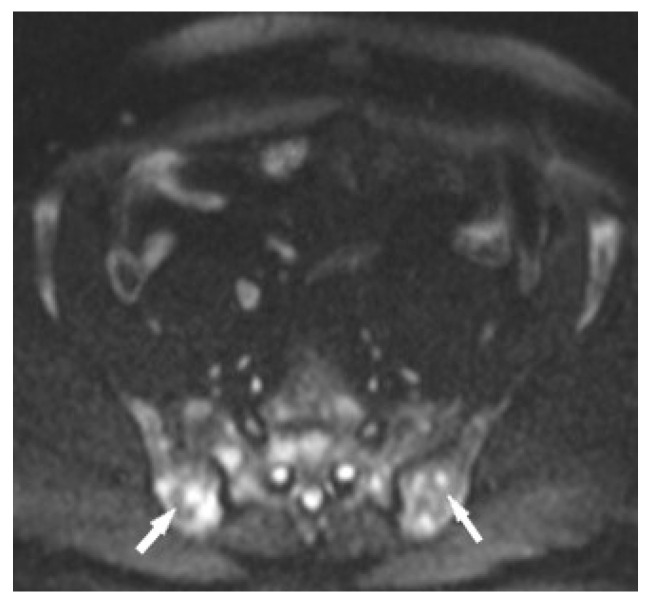
MRI of the pelvis, Diffusion-Weighted Imaging (DWI). Multiple bone marrow lesions with restricted diffusion (white arrows).

The specificity of MR imaging may be improved by diffusion-weighted imaging (Figure 10). Conte et al. pointed out that the Apparent Diffusion Coefficient (ADC) value of marrow sarcoidosis foci is lower compared to the ADC value of metastases, usually less than 700 µm^2^/s [21]. This study used DWI/ADC data from the whole-body MRI examination, but it seems that even with examinations limited to a single anatomical region it makes sense to include diffusion-weighted imaging as a part of the protocol. The authors suggest using diffusion images including other routine MR imaging sequences, which may avoid the patient’s administration of contrast. However, this does not change the clinical practice rules, that the suspicious lesion should be verified by histopathological analysis.

### 3.3. Muscular Involvement 

Muscle involvement in sarcoidosis may be asymptomatic or may present as acute or chronic myositis and sometimes it presents as painful nodules. Symptoms of muscle sarcoidosis are seen in only 1% of patients, with chronic myopathy being the most common form of the disease [20]. Gallium scintigraphy and magnetic resonance imaging are good modalities to assess muscle involvement [16]. The use of FDG PET-CT is also described [20]. Fatty atrophy and consequent muscle weakness are common complications of muscle sarcoidosis.

## 4. Treatment of Bone Sarcoidosis

The optimal therapy for bone sarcoidosis is not known and there is no consensus concerning the treatment of osseous involvement in the disease. Neither ATS nor the European Respiratory Society (ERS) nor the British Thoracic Society (BTS) sarcoidosis guidelines specify the best treatment for bone localizations [1,22,23]. As bone involvement is often a part of multisystem disease, many patients receive therapy for other organ manifestations [24]. 

Sarcoidosis frequently undergoes spontaneous regression without causing any permanent damage to the affected organs [25]. Spontaneous resolution of vertebral sarcoidosis documented by radiologic examination (X-ray and MR imaging) has been described in small number of cases by Rahmuni et al. and Johnson et al. [26,27]. 

Asymptomatic patients probably do not require treatment, although the indications for therapeutic interventions are not well defined. Treatment in sarcoidosis with osseous involvement is usually indicated in case of uncontrolled pain, stiffness, or bone destruction. It is not known whether early therapy can affect the course of the disease.

Non-steroidal anti-inflammatory drugs (NSAIDs) can be effective for symptoms relief. Glucocorticoid (GCS) oral therapy is the most frequently used first line treatment, mostly prednisone 20–40 mg, gradually reduced to 10 mg daily—the dosage is adjusted according to the clinical response. GCSs can decrease pain, but they do not normalize the bone abnormalities. Moreover, prolonged use of corticosteroids may increase the risk of osteoporosis. A careful follow-up with bone mineral densitometry and determination of calcium metabolism must be performed during steroid therapy [8,28]. 

For refractory cases of bone sarcoidosis, steroid-sparing agents may need to be added. Methotrexate (MTX) 15 mg weekly, azathioprine (AZA) 50–100 mg twice daily, and tumor necrosis factor (TNF) alpha inhibitors are the most common. Conventional synthetic disease-modifying antirheumatic drugs (csDMARDs) are used as the third line of therapy [29]. MTX used alone has been reported to be useful for the treatment of osseous sarcoidosis [12,30]. The addition of MTX to GCS in first line therapy could act as a steroid-sparing agent to reduce toxicity in bone sarcoidosis and in treatment of variety of organ symptoms [31,32]. AZA may also be effectively used as steroid-sparing agent for pulmonary and extra-pulmonary manifestation of sarcoidosis [30,33]. Only individual cases of bone sarcoidosis treated with TNF-alfa inhibitors (infliximab, adalimumab) have been available from the literature. Anti-TNF-alfa antibodies should be considered in cases of patients with vertebral sarcoidosis and aggressive disease, especially in those who fail first line therapy [34]. Antimalarial drug hydroxychloroquine may be useful in therapy of musculoskeletal disease, and it is an attractive option because of its relative safety [11]. Cyclosporine A has been successfully used [35]. 

Indications for operative interventions in osseous sarcoidosis include irreversible bone pain, neurological involvement, and radiographic evidence of severe cortical destruction such as spinal instability [36]. For patients with isolated osseous sarcoidosis lesions without a prior diagnosis of sarcoidosis, an orthopedic surgeon should perform a thorough evaluation for extra-osseous manifestation of the disease [36]. When the bone lesions occur in the absence of typical pulmonary and extra-pulmonary features of sarcoidosis, bone biopsy is needed to demonstrate the presence of pathologic features of sarcoidosis such as non-caseating granuloma [8]. 

In an American study of 24 patients with bone sarcoidosis, who had the median follow-up of 5 years, investigators found favorable clinical course in most cases [37]. After initial diagnosis, most patients with osseous sarcoidosis were either stable or improved as measured by symptoms, musculoskeletal/chest imaging, and pulmonary function tests. The distribution and number of osseous sarcoid lesions had no impact on clinical outcomes. Patients who were treated also had a favorable response [37]. The rarity of sarcoidosis, particularly osseous sarcoidosis, does not allow for the easy construction of new randomized clinical trials that would evaluate the efficacy and safety of GCSs and other immunosuppressive drugs in cases of bone involvement in sarcoidosis.

## 5. Clinical Vignette

A 34-year-old man was admitted to the pulmonary department due to non-productive cough, pain and swelling of the knee and ankle joints, and increased body temperature to 39 °C for 10 days a few weeks before admission. Ambulatory chest computed tomography (CT) revealed bilateral hilar lymphadenopathy. 

On admission, he was feverless, blood pressure (BP) was 115/90 mmHg, heart ratio (HR)—97/min, saturation O2—98%. The physical examination was normal, joint swellings subsided. Laboratory test revealed white blood cell count (WBC)—5 × 10^9^/L (normal, 4.23–9.07), neutrophils count—3.21 × 10^9^/L (normal, 1.78–5.38), C-reactive protein (CRP)—11.4 mg/L (normal <10), erythrocyte sedimentation rate (ESR)—21 (normal <8 mm/h), and angiotensin-converting enzyme level (ACE)—77.3 IU/L (normal, 8.0–52.0). During the six-minute walking test (6 MWT) the patient covered 576 m, without desaturation. Body plethysmography showed a mild degree of bronchial obstruction and a mild decrease in lung diffusion capacity for carbon monoxide (TLCO) (Table 1). 

Bronchoscopy with bronchial biopsy and an endoscopic ultrasound–guided lymph node biopsy (EBUS-TBNA) were performed. Pathologic examination of bronchial mucosa and lymph node biopsies were inconclusive. Broncho-alveolar lavage fluid (BALF) revealed increased lymphocytes count (43% of cell population), with a high CD4/CD8 index (9.96). Ziehl–Neelsen smears and cultures of bronchial washings for tuberculosis and other pathogenic organisms were negative. Finally, stage I sarcoidosis without pathologic confirmation was diagnosed. The patient did not receive any treatment. 

Subsequent chest CT showed partial regression of nodal lesions and the presence of small parenchymal nodules in the lungs. During the further follow-up, the patient reported pain in the lumbar spine and paresthesia of lower limbs. X-ray examinations of the spine did not show any evident changes. Laboratory examinations revealed normal serum calcium level and increased calcium level in a 24-h urine test (19.2 mmol/L, n: 2.5–7.5). Abdominal CT showed spleen enlargement with focal lesions suggestive of sarcoid granulomas, calcifications in the left kidney, as well as enlarged abdominal lymph nodes. Chest CT revealed progression of nodal and parenchymal lesions. An MRI scan of the lumbar spine and pelvis was performed. 

**Figure 11:**
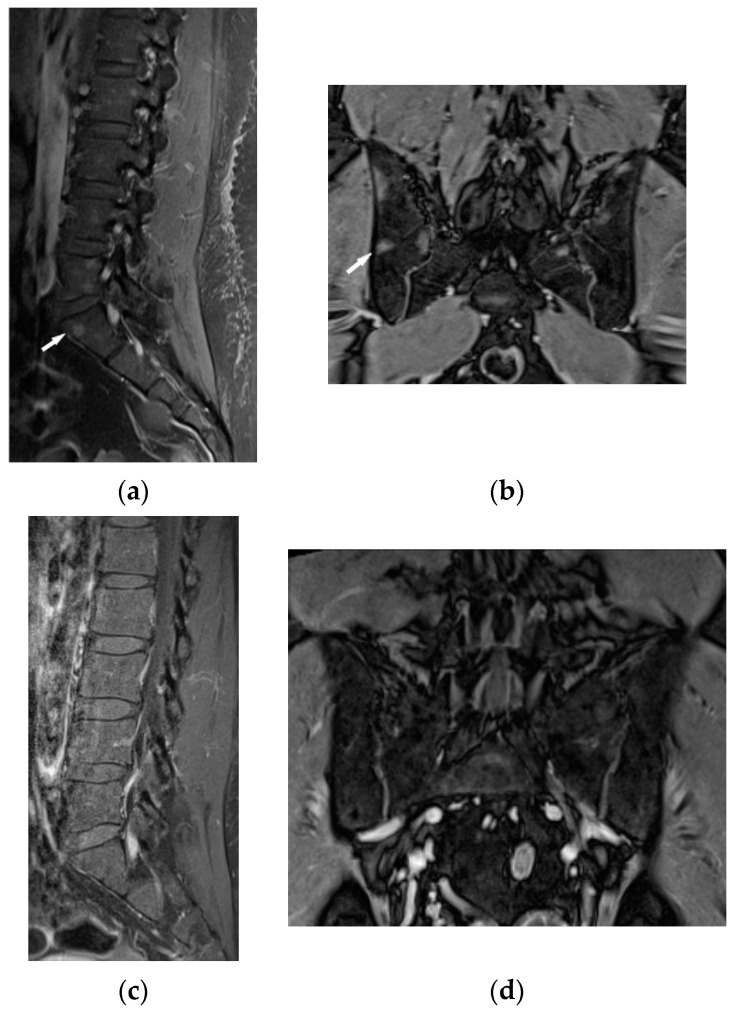
34-year-old patient. T1-weighted images, contrast enhanced, with fat saturation of the lumbar spine and pelvis. Multiple enhanced bone marrow lesions in the vertebral bodies ((**a**), white arrow) and in the pelvis at the level of the sacroiliac joints ((**b**), white arrow). Complete regression of the lesions after the treatment in the vertebral bodies (**c**) and pelvis (**d**).

The lumbar spine MRI revealed protrusion of an intervertebral disc with compression on the dural sac and nerve roots, which presumably caused the symptoms. Moreover, the images of lumbar vertebrae and pelvis showed disseminated, small enhancing lesions (Figure 11a,b), which were isointense on T1- and T2-weighted images, hyperintense on TIRM images and revealed restricted water diffusion. The MRI appearance of the lesions was nonspecific. However, the radiologist interpreting the results had access to clinical data so the differential diagnosis took into account both sarcoidosis and metastases.

It was decided to repeat bronchoscopy. Pathological confirmation of sarcoidosis was obtained from a biopsy of the bronchial mucosa (non-necrotizing granulomas and multinucleated giant cells). To exclude other than sarcoidosis changes in the bones and bone marrow, the patient was consulted in hematology and orthopedic medical centers. Iliac bone biopsy revealed multinucleated giant cells and epithelioid granulomas without necrosis. In bone fragment-containing bone marrow, the image was suggestive of sarcoid granulomas as well. Due to confirmation of sarcoidosis with bone and bone marrow involvement and progression of lung disease, prednisone at a dose of 30 mg per day and methotrexate at a dose of 20 mg per week was implemented. As a result of treatment, almost complete regression of lung disease in the control chest CT, and normalization of respiratory parameters, was observed. Normalization of calcium level in 24-h urine test (Table 1), and regression of enlarged lymphatic abdominal nodes were also noted. Control MR imaging showed almost complete regression of intramedullary foci in lumbar vertebrae and complete regression of intramedullary foci in the pelvic bone elements (Figure 11c,d). Therefore, the dose of prednisone was gradually reduced to 10 mg per day and methotrexate to 10 mg per week. 

## 6. Summary

Musculoskeletal involvement in the course of sarcoidosis is increasingly recognized due to implementation of modern imaging techniques. The most troublesome situations concern the patients with focal bone disease, recognized in patients without the signs of pulmonary sarcoidosis. Tissue biopsy is often required to differentiate between bone sarcoidosis and metastatic neoplastic disease because MR images of bone lesions are nonspecific, mandating differentiation between sarcoidosis foci and metastatic lesions or multiple myeloma. Modern imaging techniques facilitate tissue sampling and enable monitoring of the disease course. Although the spontaneous regression of bone sarcoidosis is possible, some patients require immunosuppressive therapy to achieve the remission of the disease. 

## Figures and Tables

**Table 1:** Baseline and follow-up plethysmography, TLCO, 6 MWT and calcium secretion results in patients with bone sarcoidosis.

Parameter	FVC (% Pred.)	FEV1 (% Pred.)	Tiff.	TLCO (% Pred)	Ca Urine (mmol/24 h)	6 MWD (m)	Sat 1 (%)	Sat 2 (%)
Initial	89.7	78.9	0.7	80.2	4.1	576	97	97
At progression	86.9	76.5	0.7	74.1	19.2	632	96	96
Post-treatment	87.4	83.5	0.75	85.8	2.5	648	98	97

Ca—calcium, FVC—forced vital capacity, FEV1—forced expiratory volume in 1 second, TLCO—lung diffusion capacity for carbon monoxide, 6 MWT—six minutes’ walk test, sat 1—pre-test oxygen saturation, sat 2—post-test oxygen saturation, % pred—percentage of predicted.

## Data Availability

Data is contained within the article.
